# Supplementary material for: Higher income individuals are more generous when local economic inequality is high
Source: PLoS One. 2023 Jun 14;18(6):e0286273. doi: 10.1371/journal.pone.0286273 (PMC10266655; doi:10.1371/journal.pone.0286273)
Supplement: S1 File — This file provides additional tables—descriptive statistics and robustness checks. (PDF) [file pone.0286273.s001.pdf]

Supporting information

S1 Table. Descriptive statistics for US sample

Statistic	N	Mean	St. Dev.	Min	Pctl(25)	Pctl(75)	Max
Charity Volume	152,093	0.09	0.15	0.00	0.00	0.13	1.00
Charity percent	79,775	0.01	0.01	0.0001	0.005	0.01	0.59
Gini (ZIP)	133,870	0.43	0.06	0.05	0.39	0.46	0.77
Median income	133,762	61,410.36	25,638.16	9,570.00	44,672.00	71,439.00	250,001.00
Population density (pop / sqkm)	133,964	0.66	2.24	0.0001	0.02	0.52	58.90
White (%)	133,964	0.81	0.20	0.00	0.74	0.96	1.00
Poor (%)	133,916	0.05	0.03	0.00	0.03	0.07	0.44
College degree (%)	133,964	0.18	0.12	0.00	0.10	0.23	0.86
Age 65+ (%)	133,964	0.17	0.07	0.00	0.13	0.20	0.88
Age less than 18 (%)	133,964	0.22	0.05	0.00	0.19	0.25	0.48

S2 Table. Descriptive statistics for UK sample

Statistic	N	Mean	St. Dev.	Min	Pctl(25)	Pctl(75)	Max
Charity given	22,820	1.00	0.04	0.00	1.00	1.00	1.00
Charity percent	22,775	0.67	2.17	0.00	0.07	0.53	92.90
Gini (MSOA)	21,373	0.22	0.06	0.07	0.18	0.26	0.51
Income	22,865	50,951.23	36,159.15	0.00	25,487.16	67,176.00	1,032,787.00
Economic segregation	21,373	0.27	0.10	0.03	0.20	0.32	0.83
Age	22,861	51.33	17.92	16.00	38.00	65.00	102.00
Female	22,865	0.57	0.49	0	0	1	1
Degree	22,549	0.44	0.50	0.00	0.00	1.00	1.00
White	22,822	0.84	0.37	0.00	1.00	1.00	1.00
Employed	22,837	0.59	0.49	0.00	0.00	1.00	1.00
Married	22,824	0.58	0.49	0.00	0.00	1.00	1.00
Religious	22,839	0.57	0.49	0.00	0.00	1.00	1.00
Conservatives	22,865	0.29	0.45	0	0	1	1
Labour	22,865	0.33	0.47	0	0	1	1
Liberal Democrat	22,865	0.07	0.26	0	0	0	1
Other political party	22,865	0.30	0.46	0	0	1	1
Population density	21,373	32.15	34.98	0.02	5.50	44.50	247.20
Median house value	21,373	256,571.90	152,621.10	53,000.00	148,000.00	323,000.00	2,198,000.00

S3 Table. Robustness checks, US ZIP-level inequality

	Dependent variable:							
	Top 5% concentration	Gini 2007-2011	Population < 50k	Population >= 50k	Top 5% concentration	Gini 2007-2011	Population < 50k	Population >= 50k
	Percent of income group donating to charity				Average percent of income donated			
	(1)	(2)	(3)	(4)	(5)	(6)	(7)	(8)
Gini	0.134***	0.124***	0.157***	0.055***	-0.009	-0.034***	-0.048***	0.026
	(0.007)	(0.008)	(0.009)	(0.018)	(0.006)	(0.006)	(0.007)	(0.025)
$37,500	1.371***	1.370***	1.367***	1.376***	0.464***	0.464***	0.423***	0.774***
	(0.002)	(0.002)	(0.002)	(0.004)	(0.006)	(0.006)	(0.006)	(0.015)
$62,500	2.336***	2.334***	2.314***	2.405***	0.869***	0.868***	0.817***	1.317***
	(0.002)	(0.002)	(0.002)	(0.004)	(0.005)	(0.005)	(0.006)	(0.015)
$87,500	2.765***	2.761***	2.714***	2.954***	1.036***	1.035***	0.977***	1.569***
	(0.002)	(0.002)	(0.002)	(0.004)	(0.006)	(0.006)	(0.006)	(0.015)
$150,000	3.342***	3.338***	3.291***	3.544***	1.359***	1.354***	1.297***	1.899***
	(0.002)	(0.002)	(0.002)	(0.004)	(0.005)	(0.005)	(0.006)	(0.015)
$200,000+	4.275***	4.272***	4.239***	4.446***	1.896***	1.892***	1.846***	2.297***
	(0.002)	(0.002)	(0.002)	(0.004)	(0.006)	(0.006)	(0.006)	(0.015)
Gini:$37,500	-0.071***	-0.046***	-0.036***	-0.009**	-0.018***	0.003	0.021***	0.039**
	(0.002)	(0.002)	(0.002)	(0.004)	(0.006)	(0.006)	(0.006)	(0.017)
Gini:$62,500	-0.112***	-0.071***	-0.061***	0.002	-0.007	0.031***	0.046***	0.063***
	(0.002)	(0.002)	(0.002)	(0.004)	(0.006)	(0.006)	(0.006)	(0.017)
Gini:$87,500	-0.065***	-0.015***	-0.004**	0.079***	0.025***	0.064***	0.084***	0.113***
	(0.002)	(0.002)	(0.002)	(0.004)	(0.006)	(0.006)	(0.006)	(0.017)
Gini:$150,000	-0.029***	0.021***	0.028***	0.107***	0.032***	0.068***	0.080***	0.115***
	(0.002)	(0.002)	(0.002)	(0.004)	(0.006)	(0.006)	(0.006)	(0.017)
Gini:$200,000+	0.119***	0.125***	0.139***	0.182***	0.104***	0.140***	0.141***	0.208***
	(0.002)	(0.002)	(0.002)	(0.004)	(0.006)	(0.006)	(0.006)	(0.018)
Median income	0.135***	0.123***	0.144***	0.148***	0.174***	0.175***	0.174***	0.091**
	(0.014)	(0.014)	(0.015)	(0.029)	(0.008)	(0.008)	(0.008)	(0.036)
Ln(Population)	-0.030***	-0.036***	-0.038***	-0.111***	-0.041***	-0.043***	-0.048***	-0.034***
	(0.007)	(0.007)	(0.007)	(0.016)	(0.004)	(0.004)	(0.006)	(0.007)
White (%)	-0.363***	-0.364***	-0.363***	-0.120***	-0.142***	-0.140***	-0.144***	-0.148***
	(0.008)	(0.009)	(0.009)	(0.012)	(0.005)	(0.005)	(0.005)	(0.012)
Poor (%)	-0.204***	-0.224***	-0.262***	-0.155***	0.043***	0.033***	0.030***	-0.101***
	(0.010)	(0.011)	(0.012)	(0.024)	(0.005)	(0.006)	(0.006)	(0.028)
25+ with college degree (%)	0.370***	0.386***	0.379***	-0.043	-0.094***	-0.107***	-0.112***	0.013
	(0.012)	(0.012)	(0.013)	(0.026)	(0.007)	(0.007)	(0.007)	(0.030)
Age 65+ (%)	-0.001	0.006	0.002	0.044***	0.186***	0.183***	0.180***	0.136***
	(0.008)	(0.008)	(0.009)	(0.012)	(0.004)	(0.004)	(0.005)	(0.019)
Age less than 18 (%)	-0.009	0.008	-0.008	0.022	0.028***	0.021***	0.019***	0.100***
	(0.009)	(0.009)	(0.009)	(0.015)	(0.005)	(0.005)	(0.005)	(0.016)
Constant	-5.022***	-5.076***	-5.062***	-4.566***	-5.243***	-5.228***	-5.183***	-5.723***
	(0.041)	(0.042)	(0.044)	(0.162)	(0.023)	(0.023)	(0.023)	(0.173)
State fixed effect	Y	Y	Y	Y	Y	Y	Y	Y
Random effect level	ZIP-code	ZIP-code	ZIP-code	ZIP-code	ZIP-code	ZIP-code	ZIP-code	ZIP-code
Observations	124,802	128,101	122,712	5,389	78,220	78,732	73,391	5,341
Log Likelihood	-1,017,422.000	-1,035,171.000	-964,688.500	-64,951.210	-53,249.720	-54,117.250	-50,953.720	-2,284.816
Akaike Inf. Crit.	2,034,984.000	2,070,481.000	1,929,517.000	130,022.400	106,641.400	108,376.500	102,049.400	4,691.632

Note: *p<0.1; **p<0.05; ***p<0.01

Coefficients of continuous independent variables are standardised (i.e. mean is grand-centred, standard deviation = 1). Standard errors are in parentheses. Data sourced from [1] and [2].

S4 Table. US state and county-level inequality

	Dependent variable:			
	State-level	County-level	State-level	County-level
	Percent of income group donating to charity		Average percent of income donated	
	(1)	(2)	(3)	(4)
Gini	0.009	0.052***	-0.031	-0.105***
	(0.074)	(0.003)	(0.075)	(0.017)
$17,500	0.852***	0.844***	0.334***	-0.025
	(0.004)	(0.004)	(0.047)	(0.018)
$37,500	1.835***	1.814***	0.876***	0.320***
	(0.004)	(0.003)	(0.047)	(0.018)
$62,500	2.816***	2.790***	1.382***	0.740***
	(0.003)	(0.003)	(0.047)	(0.017)
$87,500	3.290***	3.262***	1.562***	0.899***
	(0.003)	(0.003)	(0.047)	(0.018)
$150,000	3.915***	3.866***	1.991***	1.410***
	(0.003)	(0.003)	(0.047)	(0.017)
$200,000+	5.077***	4.940***	2.905***	2.203***
	(0.003)	(0.003)	(0.047)	(0.018)
Gini:$17,500	-0.118***	-0.114***	-0.003	0.027
	(0.004)	(0.003)	(0.047)	(0.019)
Gini:$37,500	-0.059***	-0.044***	0.119**	0.171***
	(0.003)	(0.003)	(0.047)	(0.018)
Gini:$62,500	-0.064***	-0.050***	0.085*	0.202***
	(0.003)	(0.003)	(0.047)	(0.018)
Gini:$87,500	-0.024***	-0.004	0.069	0.210***
	(0.003)	(0.003)	(0.047)	(0.018)
Gini:$150,000	-0.042***	-0.017***	-0.024	0.162***
	(0.003)	(0.003)	(0.047)	(0.018)
Gini:$200,000+	-0.111***	0.002	-0.033	0.151***
	(0.003)	(0.003)	(0.047)	(0.018)
Median income	0.036	0.272***	-0.236**	-0.00002
	(0.127)	(0.002)	(0.118)	(0.013)
Ln(Population)	0.128***	0.034***	0.058	-0.225***
	(0.041)	(0.001)	(0.038)	(0.007)
White (%)	-0.192***	-0.302***	-0.218***	-0.293***
	(0.052)	(0.001)	(0.048)	(0.006)
Poor (%)	0.114	0.036***	-0.012	-0.019*
	(0.111)	(0.003)	(0.103)	(0.011)
25+ with college degree (%)	0.344***	0.039***	0.308***	0.162***
	(0.078)	(0.001)	(0.072)	(0.009)
Age 65+ (%)	0.159**	0.116***	0.033	0.099***
	(0.069)	(0.001)	(0.064)	(0.008)
Age less than 18 (%)	0.204***	0.035***	0.214***	0.120***
	(0.070)	(0.002)	(0.065)	(0.007)
Constant	-5.286***	-5.745***	-6.486***	-5.929***
	(0.032)	(0.013)	(0.043)	(0.017)
State fixed effect	N	Y	N	Y
Random effect level	State	County	State	County
Observations	364	21,816	364	15,795
Log Likelihood	-100,432.500	-359,506.800	-76.174	-11,080.460
Akaike Inf. Crit.	200,909.000	719,057.600	198.348	22,206.930

Note: *p<0.1; **p<0.05; ***p<0.01

Continuous independent variables are standardised (i.e. mean is grand-centred, standard deviation = 1). Standard errors are in parentheses. The income group reference category is $1-$9,999. Inequality data from [1], charitable donation and income group from [2].

S5 Table. Charitable donation amount, UK MSOA-level inequality

	Dependent variable:	
	Amount donated to charity (£)	
	(1)	(2)
Gini	0.077***	0.075***
	(0.012)	(0.012)
Income (£/year)	0.189***	0.185***
	(0.011)	(0.011)
Gini:Income		0.026***
		(0.008)
Economic segregation	-0.025**	-0.025**
	(0.012)	(0.012)
Age	0.021***	0.021***
	(0.001)	(0.001)
Male	0.086***	0.086***
	(0.016)	(0.016)
Degree	0.462***	0.462***
	(0.019)	(0.019)
White	-0.138***	-0.142***
	(0.033)	(0.033)
Employed	0.235***	0.236***
	(0.021)	(0.021)
Married	0.134***	0.133***
	(0.020)	(0.020)
Religious	0.316***	0.316***
	(0.020)	(0.020)
Labour	-0.055**	-0.055**
	(0.024)	(0.024)
Liberal Democrat	0.139***	0.139***
	(0.036)	(0.036)
Other political party	-0.158***	-0.157***
	(0.026)	(0.026)
Population density	0.090***	0.087***
	(0.015)	(0.015)
Median house value	0.088***	0.085***
	(0.018)	(0.018)
Constant	2.825***	2.826***
	(0.076)	(0.076)
Region fixed effect	Y	Y
Random effect level	MSOA and Household	MSOA and Household
Observations	20,860	20,860
Log Likelihood	-34,671.510	-34,670.680
Akaike Inf. Crit.	69,401.030	69,401.370
Note:	*p<0.1; **p<0.05; ***p<0.01	

Coefficients of continuous independent variables are standardised (i.e. mean is grand-centred, standard deviation = 1). Standard errors are in parentheses. Inequality data from [3] and survey data from [4].

S6 Table. Top 1% inequality regression, UK MSOA-level inequality

	Dependent variable:	
	Donated to charity?	Percent donated to charity
	(1)	(2)
Top 1% share	0.007**	0.061***
	(0.003)	(0.011)
Income (£/year)	0.037***	-0.372***
	(0.003)	(0.011)
Top 1% share:Income	-0.003	0.024***
	(0.003)	(0.009)
Economic segregation	-0.004	-0.024**
	(0.003)	(0.012)
Age	0.004***	0.025***
	(0.0002)	(0.001)
Male	-0.064***	0.078***
	(0.005)	(0.017)
Degree	0.106***	0.435***
	(0.006)	(0.019)
White	0.051***	-0.159***
	(0.009)	(0.033)
Employed	0.073***	0.131***
	(0.006)	(0.022)
Married	0.035***	-0.025
	(0.006)	(0.021)
Religious	0.068***	0.318***
	(0.006)	(0.020)
Labour	-0.018***	-0.059**
	(0.007)	(0.024)
Liberal Democrat	0.017	0.128***
	(0.011)	(0.037)
Other political party	-0.072***	-0.148***
	(0.007)	(0.026)
Population density	-0.0002	0.102***
	(0.004)	(0.015)
Median house value	0.032***	0.114***
	(0.005)	(0.017)
Constant	0.370***	-3.075***
	(0.021)	(0.078)
Region fixed effect	Y	Y
Random effect level	MSOA and Household	MSOA and Household
Observations	31,390	20,860
Log Likelihood	-19,054.350	-35,086.020
Akaike Inf. Crit.	38,168.690	70,232.040
Note:	*p<0.1; **p<0.05; ***p<0.01	

Coefficients of continuous independent variables are standardised (i.e. mean is grand-centred, standard deviation = 1). Standard errors are in parentheses. Inequality data from [3] and survey data from[4].

S7 Table. UK LSOA and Local Authority District inequality

	Dependent variable:			
	LSOA	LAD	LSOA	LAD
	Donated to charity?		Percent donated to charity	
	(1)	(2)	(3)	(4)
Gini	0.062^{***}	0.026	0.076^{***}	0.042^{**}
	(0.017)	(0.026)	(0.012)	(0.018)
Income (£/year)	0.209^{***}	0.236^{***}	-0.381^{***}	-0.354^{***}
	(0.018)	(0.018)	(0.011)	(0.011)
Gini:Income	0.004	0.042^{***}	0.027^{***}	0.025^{**}
	(0.016)	(0.016)	(0.009)	(0.010)
Economic segregation	0.010	-0.074^{***}	-0.024^{**}	-0.031
	(0.016)	(0.028)	(0.011)	(0.019)
Age	0.021^{***}	0.021^{***}	0.024^{***}	0.025^{***}
	(0.001)	(0.001)	(0.001)	(0.001)
Male	-0.323^{***}	-0.313^{***}	0.076^{***}	0.073^{***}
	(0.027)	(0.028)	(0.017)	(0.017)
Degree	0.551^{***}	0.585^{***}	0.426^{***}	0.458^{***}
	(0.030)	(0.031)	(0.019)	(0.020)
White	0.261^{***}	0.310^{***}	-0.183^{***}	-0.161^{***}
	(0.046)	(0.046)	(0.033)	(0.034)
Employed	0.329^{***}	0.320^{***}	0.135^{***}	0.114^{***}
	(0.031)	(0.032)	(0.022)	(0.022)
Married	0.172^{***}	0.170^{***}	-0.026	-0.030
	(0.031)	(0.031)	(0.021)	(0.021)
Religious	0.364^{***}	0.363^{***}	0.320^{***}	0.302^{***}
	(0.031)	(0.032)	(0.020)	(0.021)
Labour	-0.091^{**}	-0.110^{***}	-0.051^{**}	-0.063^{**}
	(0.038)	(0.039)	(0.024)	(0.025)
Liberal Democrat	0.123^{*}	0.125^{*}	0.130^{***}	0.134^{***}
	(0.064)	(0.066)	(0.037)	(0.038)
Other political party	-0.340^{***}	-0.351^{***}	-0.142^{***}	-0.162^{***}
	(0.039)	(0.040)	(0.026)	(0.027)
Population density	0.010	-0.015	0.091^{***}	0.081^{***}
	(0.019)	(0.031)	(0.014)	(0.021)
Median house value	0.170^{***}	0.046	0.127^{***}	0.010
	(0.024)	(0.048)	(0.015)	(0.033)
Region fixed effect	Y	Y	Y	Y
Random effect level	LSOA and Household	LAD and Household	LSOA and Household	LAD and Household
Observations	31,348	29,686	20,836	19,689
Log Likelihood	-18,113.500	-17,219.210	-35,031.780	-33,108.630
Akaike Inf. Crit.	36,285.000	34,496.420	70,123.560	66,277.260

Note: $^{*}p<0.1$; $^{**}p<0.05$; $^{***}p<0.01$

Coefficients of continuous independent variables are standardised (i.e. mean is grand-centred, standard deviation = 1). Standard errors are in parentheses. Inequality data from [3] and survey data from [4].

S8 Table. Restricted sample regression, respondents who have never moved, UK MSOA-level inequality

	Dependent variable:	
	Donated to charity?	Percent donated to charity
	(1)	(2)
Gini	0.024*	0.159***
	(0.013)	(0.048)
Income (£/year)	0.024**	-0.530***
	(0.011)	(0.040)
Gini:Income	0.004***	0.024***
	(0.001)	(0.003)
Economic segregation	0.004	-0.055
	(0.012)	(0.045)
Age	-0.071***	0.016
	(0.020)	(0.076)
Male	0.084***	0.412***
	(0.024)	(0.086)
Degree	0.021	-0.396***
	(0.031)	(0.117)
White	0.076***	0.402***
	(0.022)	(0.083)
Employed	-0.002	0.291***
	(0.031)	(0.111)
Married	0.075***	0.382***
	(0.025)	(0.094)
Religious	-0.008	-0.114
	(0.033)	(0.118)
Labour	0.120**	0.248
	(0.060)	(0.201)
Liberal Democrat	-0.108***	-0.163
	(0.032)	(0.118)
Other political party	0.014	0.065
	(0.012)	(0.045)
Population density	0.013	-0.135*
	(0.019)	(0.070)
Median house value	-0.006	0.032
	(0.010)	(0.037)
Constant	0.333***	-3.160***
	(0.073)	(0.279)
Region fixed effect	Y	Y
Random effect level	MSOA and Household	MSOA and Household
Observations	2,348	1,159
Log Likelihood	-1,669.907	-1,964.161
Akaike Inf. Crit.	3,399.814	3,988.322

Note: *p<0.1; **p<0.05; ***p<0.01

Coefficients of continuous independent variables are standardised (i.e. mean is grand-centred, standard deviation = 1). Standard errors are in parentheses. Inequality data from [3] and survey data from [4].

S9 Table. Volunteering regression, UK MSOA-level inequality

	Dependent variable:					
	Volunteered?			Number of hours volunteered		
	(1)	(2)	(3)	(4)	(5)	(6)
Gini	0.031***	0.016***	0.016***	-0.026*	-0.038**	-0.042**
	(0.002)	(0.003)	(0.003)	(0.015)	(0.017)	(0.017)
Income (£/year)	0.024***	0.019***	0.019***	-0.106***	-0.041***	-0.054***
	(0.002)	(0.003)	(0.003)	(0.015)	(0.016)	(0.017)
Gini:Income	0.002		-0.0001	0.032**		0.026**
	(0.002)		(0.002)	(0.013)		(0.013)
Economic segregation		-0.011***	-0.011***		0.008	0.009
		(0.003)	(0.003)		(0.017)	(0.017)
Age		-0.001***	-0.001***		0.002**	0.002**
		(0.0001)	(0.0001)		(0.001)	(0.001)
Male		-0.002	-0.002		0.068**	0.068**
		(0.004)	(0.004)		(0.029)	(0.029)
Degree		0.092***	0.092***		0.056*	0.056*
		(0.005)	(0.005)		(0.030)	(0.030)
White		0.044***	0.044***		-0.0005	-0.001
		(0.007)	(0.007)		(0.050)	(0.050)
Employed		-0.070***	-0.070***		-0.256***	-0.253***
		(0.005)	(0.005)		(0.033)	(0.033)
Married		-0.005	-0.005		-0.047	-0.045
		(0.005)	(0.005)		(0.033)	(0.033)
Religious		0.059***	0.059***		0.002	0.004
		(0.005)	(0.005)		(0.032)	(0.032)
Labour		-0.007	-0.007		0.038	0.037
		(0.006)	(0.006)		(0.038)	(0.038)
Liberal Democrat		0.051***	0.051***		0.099*	0.098*
		(0.009)	(0.009)		(0.052)	(0.051)
Other political party		-0.013**	-0.013**		0.104**	0.102**
		(0.006)	(0.006)		(0.041)	(0.041)
Population density		-0.005	-0.005		-0.019	-0.020
		(0.003)	(0.003)		(0.020)	(0.020)
Median house value		0.027***	0.027***		-0.013	-0.014
		(0.004)	(0.004)		(0.023)	(0.023)
Constant	0.188***	0.197***	0.197***	2.227***	2.240***	2.244***
	(0.002)	(0.017)	(0.017)	(0.015)	(0.114)	(0.114)
Region fixed effect	N	Y	Y	N	Y	Y
Random effect level	MSOA and Household	MSOA and Household	MSOA and Household	MSOA and Household	MSOA and Household	MSOA and Household
Observations	34,659	33,701	33,701	4,627	4,563	4,563
Log Likelihood	-15,787.910	-15,076.890	-15,082.200	-6,405.240	-6,315.042	-6,316.315
Akaike Inf. Crit.	31,589.830	30,211.780	30,224.400	12,824.480	12,688.080	12,692.630

Note: *p<0.1; **p<0.05; ***p<0.01

Coefficients of continuous independent variables are standardised (i.e. mean is grand-centred, standard deviation = 1). Standard errors are in parentheses. Inequality data from [3] and survey data from [4].

References

1. US Census Bureau. *American Community Survey. Gini index estimates (table B19083). [Data collection]*. Available at factfinder.census.gov/faces/nav/jsf/pages/index.xhtml; 2021.
2. IRS. *Internal revenue service tax. [Data collection]*. Available at https://www.irs.gov/statistics/soi-tax-stats-county-data-2018; 2018.
3. Suss JH. Measuring local, salient economic inequality in the UK. Environment and Planning A: Economy and Space. 2023.
4. University of Essex Institute for Social and Economic Research. *Understanding Society: Waves 1-10, 2009-2019 and harmonised BHPS: Waves 1-18, 1991-2009. [Data collection]*. Vols. 13th Edition. UK Data Service, SN: 6614; 2020. DOI: http://doi.org/10.5255/UKDA-SN-6614-18
