# Supplementary material for: Higher income individuals are more generous when local economic inequality is high
Source: PLoS One. 2023 Jun 14;18(6):e0286273. doi: 10.1371/journal.pone.0286273 (PMC10266655; doi:10.1371/journal.pone.0286273)
Supplement: Attachment — Submitted filename: revision_reply.docx [file pone.0286273.s002.docx]

**Editors of PLOS ONE**

Dear Editors and Reviewers,

I greatly appreciate the comments for my article entitled ‘Higher income individuals are more generous when local economic inequality is high’. These are constructive and fair. Many thanks to the editor and reviewers for the time and effort they have spent reading and commenting.

First and foremost, I was pleased to hear that the article could be of interest for the journal. Specifically,

The academic editor said:

- *The reviewers and I found a number of positive aspects in the research. All of us agreed that you have tackled a timely and highly relevant question regarding the association between inequality and prosocial behavior*

Reviewer 1 said:

- *I really liked the paper, it is interesting and it provides an added value to the literature.*

Reviewer 2 said:

- *The manuscript addresses a highly relevant topic of current debate: the consequences of income inequality on pro-social behaviour*

Reviewer 3 said:

- *sophisticated analyses have been conducted using multi-level modeling*

It is clear, however, that I could strengthen the paper substantially before publication and I am grateful to the reviewers for pointing out the areas requiring further development. In response to the comments, I have improved the manuscript as follows (my point-by-point response to comments in **bold**):

Academic editor:

I echo the concern raised by Reviewer 1 about the definition of inequality, which is somewhat neglected in the manuscript. The term inequality may mean different things to different people and in different contexts. I think engaging more with the definition discussion would certainly benefit the manuscript.

**This omission has now been rectified, thank-you for noting this. I have made clear in the introduction and methods section that I measure inequality (in the baseline analysis) using the Gini coefficient – see first paragraph p. 4 for the first mention: “*This paper tests this hypothesis using granular data on local economic inequality – measured by the Gini coefficient – for the US and UK*”.**

**Also, as robustness checks, I examine whether the results are sensitive to alternative measures of inequality (share of top 1% for UK sample, top 5% for US sample).**

I also echo the serious concern raised by Reviewer 2 (see the first paragraph of the comment). It is unclear what the significance of the findings are because it seems that this research does not emphasize a theoretical advance. I struggle to find enough of a theoretical advancement and the manuscript’s theoretical contribution seems to be limited.

**Thank-you, I agree with this point – the draft was far too light on theoretical motivation and lacked a clear focus. I have now provided substantial additions – I’ve written a new section (“Background”, starting p.5) immediately after the introduction and before the data section to provide a comprehensive review of the relevant literature, and to be more specific about the theoretical justification and motivation for analysing the moderating effect of local inequality on the relationship between income and pro-social behaviour.**

I also like you to account and provide more justification in the introduction for why you focused on specific cultural contexts (i.e., the US and The UK), for the selection of variables, samples, use of methods and the statistical analyses. I think that this research suffers a flaw that it is not clear whether and to what extent testing your hypotheses for people in the US and the UK was valid and meaningful. You acknowledge yourself that the UK and US differ in many respects.

**Thank-you, I have now made clear why the US and UK are focus (availability of granular inequality estimates) in the introduction (first paragraph p. 4) – “*I focus on these countries and time period due to the availability of large-scale and detailed information on income, charitable giving (used as the main proxy for pro-sociality) and, crucially, spatially-granular estimates of economic inequality which are not generally available for other countries*”**

**I also note this caveat in the concluding section, as this is certainly a weakness of this analysis as you rightly point out. With more and more granular estimates of economic inequality (some from alternative sources), future research may be able to expand the analysis.**

You use mediation to test one of your hypothesis, but I worry you are not actively engaging with the assumptions of mediation modelling and ensuring that your analysis/model is theoretically valid (Spencer, Zanna & Fong, G. T, 2005 ; Bullock & Green, 2021; Götz et al., 2020; Stuart et al., 2022).

**Many thanks for highlighting this and for the references, which have been very helpful. I have decided to remove the section and discussion on mediation, as I do not feel that the analysis provides enough of a contribution to the paper overall and, as you point out, the assumptions for statistical mediation are unlikely to be met given the nature of the data.**

I also think that the structure of your manuscript at times feels quite fragmented and as Reviewer 3 points the methods appear everywhere. I recommend you to follow a standardized structure of a scientific manuscript.

**I have clarified the structure, for example confining the detail of the methods and data to that section (thereby including less detail in the introductory section). I have also provided a short summary of results in the introduction as is conventional, and included a paragraph laying out the structure at the end of the introduction (last paragraph on p.4). Finally, I move much of the description of the robustness checks to the methods section rather than have it in the results section.**

Reviewer 1:

1. Inequality is not defined. You have to check the figure to realize that the Gini coefficient is used. This should be explained in the introduction and in the data section. Introduction should also include that other measures were included in the robustness section.

**Thank-you for pointing out this omission – I have now introduced this earlier. Please see the introduction (first paragraph p.4): “*This paper tests this hypothesis using granular data on local economic inequality – measured by the Gini coefficient – for the US and UK*”**

**I have also now made clear in the methods section that the Gini coefficient is used as the inequality measure for the main analysis, but that this is varied as part of the robustness checks. In particular, I also use share of the top 1% (for UK sample) and top 5% (for US sample) as alternative measures. See first paragraph p.8: “*I obtain income inequality estimates, taken as the Gini coefficient in the baseline analysis (top 5% share of income in robustness checks), from the American Community Survey (ACS) for the five year period 2014-2018*”, and second paragraph p.9 for the UK “*As with the US sample, local inequality is taken as the Gini coefficient in the main analysis, but in robustness checks I check whether the results hold when inequality is measured as the top 1% share*”**

2. Is it possible to calculate income inequality using tax data? One challenge with the ACS is how representative is at a very disaggregated level, and the role of outliers.

**This is a good idea, unfortunately however there is no source of spatially disaggregated measures of income inequality using tax data. This has been used to measure income (and wealth) inequality at spatially aggregated levels, but I’m unaware of any granular estimates developed from this type of data.**

**However, I acknowledge that the ACS may have reliability issues at disaggregated levels given the size of the survey. To mitigate this, I remove very small ZIP areas from the dataset. In robustness checks, I also examine whether the results are consistent when looking at small and large ZIP-code areas separately (threshold defined as population below or above 50k). Please see second to last paragraph on p.8 in the methods section: “*In the main analysis, I drop ZIP-code areas with less than 1,000 residents to mitigate concerns around inequality estimates for smaller areas. In robustness checks, I further restrict the sample according to size of the ZIP-code area population (above and below a threshold of 50k residents).*”.**

**Note also that using the UK sample also alleviates this concern, since inequality measures there are based on a much larger dataset (~23 million observations, the near universe of housing values in the country) which might be considered to be more reliable.**

**Also, I carry out additional robustness checks on the US sample to address this further. First, I use the margin of error (95% level) provided for the Gini estimates, reducing the sample to only those ZIP-areas where the margin of error on the estimate is less than the 90^th^ percentile margin of error (0.1154). Second, I reduce the sample to only those ZIP-areas for which the margin of error on the Gini estimate is less than or equal to 0.05 (56% of total ZIP-areas). In both these cases, the results are unaffected. I don’t report this additional check in the paper, but you can see it in this reply below (Table 1).**

3. Figure 2 should include confidence intervals. Also, the text does not explain what the estimates mean. Is it plotting marginal effects? Odd-ratios? It is not clear. The interpretation of the estimates should be clearer in the text.

**I have replaced this figure with a table (Table 1, p.13) and clarified in the text and note below the table that the coefficients are standardised.**

The explanation of results in Study 1 and Study 2 seem different. Could it be possible to homogeneize a little bit more?

**I have now focused the paper more clearly on the moderating effect of inequality on the relationship between income and pro-social behaviour which I think accomplishes this. The results should now be clearer and are more consistent across both contexts (i.e. there is a positive moderating effect).**

4. An important aspect to discuss is how preferences evolve over time. The estimates found are not fixed, but subject to culture and social norms. Then, it is possible that the relationship between inequality and prosocial behavior was modified recently, such that an important avenue for future research is how and when these preferences change.

**This is a very good point, thank-you for raising it. I have focused on these specific countries and points in time due primarily to availability of granular estimates of economic inequality, but agree that the results may vary over time and across country. I’ve added a sentence in the introduction to acknowledge this, emphasising also the time dimension (first paragraph p. 4): “*I focus on these countries and time period due to the availability of large-scale and detailed information on income, charitable giving (used as the main proxy for pro-sociality) and, crucially, spatially-granular estimates of economic inequality which are not generally available for other countries.*”**

**And similarly I have noted this in the concluding section (last paragraph, p.18): “*Future work might provide additional evidence to support the findings here. For example, while I analyse data from two separate countries, given importance of national culture and norms for pro-social behaviours (48), future work could address whether the findings here generalise to other contexts and cultures and/or time periods.*”**

Reviewer 2:

1) Focus of study: The paper will benefit from a clear focus. At the moment the author seems to address various questions: the inequality-prosocial behaviour link, the moderation of inequality on the income-prosocial behaviour link, and a potential mediator (friendship). This is very appealing – but at the same time, all these questions/links have to be addressed adequately at a theoretical, conceptual and empirical level. From my perspective, this study (in its current state) does not meet these standards. However, I believe that after careful revision, this paper certainly has the potential to do so.

**Thank-you, I think this is a very important comment and very much agree that the paper was not adequately focused. I have substantially edited the paper to concentrate on the moderating effect of local inequality on the relationship between income and prosocial behaviour. I have not completely removed the discussion of the main effect of inequality on pro-social behaviour, as this is important in the context of the moderating effect, although this is no longer as prominent.**

**The most important change has been the addition of a new section, “Background”, immediately following the introduction to provide a more extensive literature review, provide additional theoretical motivation and content, and be more specific about the focus of the paper and hypothesis being tested.**

2) Literature review: the paper will certainly benefit from a broader overview of the literature. At the moment, the choice of the cited literature seems somewhat eclectic and its description falls short.

**Thank-you, please see response to comment above (and pp. 5-7 for the added literature review).**

3) Theory: At the theoretical level, the paper is still underdeveloped and a clear specification and justification of hypotheses is missing. Adding this, will certainly improve the value of this paper. At this point, the main argument of the author focusses on the geographic unit (which is well portrayed) – but the author does not test this empirically and does not compare different unit effect. Instead, the paper tests the specific links between economic inequality, pro-social behaviour and income groups with one geographic unit (for each country: the US and the UK) – and these links are theoretically not clearly specified.

**See comment to point 1) above and new “Background” section of the paper. In particular, the sub-section therein, “Local inequality and social contact”, provides theoretical rationale for why local inequality might moderate the relationship between income and pro-social behaviour. And key paragraph (bottom p.6): “*Taken together, this suggests that local inequality might lead relatively rich individuals to act more pro-socially. This leads to the formulation of the following local inequality hypothesis: local inequality is expected to interact positively with income for pro-social behaviour. In other words, the relationship between income and pro-social behaviour should depend on local inequality such that higher income households will be relatively more generous.*”**

**Regarding the geographic unit, the main analysis focuses on the ZIP-code area (the most disaggregated possible) in the US and the MSOA-level in the UK, and in recognition of your comment here there is additional analysis (Tables A.4 & A.7 in Annex) which varies the spatial unit of analysis for both contexts, in particular county and state for the US, and LSOA and local authority district for the UK.**

4) Measurement of Income Inequality: I fully agree with the authors’ argument that economic inequality observed at smaller geographic units, such as neighbourhood/ZIP code level, are of vital importance and even more likely to influence social behaviour – due to the mechanisms described by the author (i.e. direct experience and less biased perceptions of inequality). However, the measurement of income inequality at smaller geographic units is also more complex and more likely to be biased.

**Thank-you, this is an important comment. I agree that the ACS may have reliability issues at disaggregated levels given the size of the survey. To mitigate this, I remove very small ZIP areas from the dataset. In robustness checks, I also examine whether the results are consistent when looking at small and large ZIP-code areas separately (threshold defined as population below or above 50k). Please see second to last paragraph on p.8 in the methods section: “*In the main analysis, I drop ZIP-code areas with less than 1,000 residents to mitigate concerns around inequality estimates for smaller areas. In robustness checks, I further restrict the sample according to size of the ZIP-code area population (above and below a threshold of 50k residents).*”.**

**Note also that using the UK sample also alleviates this concern, since inequality measures there are based on a much larger dataset (~23 million observations, the near universe of housing values in the country) which might be considered to be more reliable.**

**Also, I carry out additional robustness checks on the US sample to address this further. First, I use the margin of error (95% level) provided for the Gini estimates, reducing the sample to only those ZIP-areas where the margin of error on the estimate is less than the 90^th^ percentile margin of error (0.1154). Second, I reduce the sample to only those ZIP-areas for which the margin of error on the Gini estimate is less than or equal to 0.05 (56% of total ZIP-areas). In both these cases, the results are unaffected. I don’t report this additional check in the paper, but you can see it in this reply below (Table 1).**

As the author points out correctly, segregation effects have to be addressed – not only empirically, but also theoretically (-> if there is more segregation, people may not observe and experience as much inequality as we would expect when studying consequences of inequality at larger geographic units).

**Regarding segregation, I agree with your comment. This may indeed be the reason why there are contrasting results for aggregate and local inequality effects, which I note in the concluding section. A reasonable assumption is that local inequality involves contact between rich and poor in a way that aggregate level inequality does not, but I have now more explicitly addressed this. First in the theoretical section, and then I include an additional control variable for economic segregation (available only for UK sample) to address this.**

**In the theory section (Footnote 5, p.7): “*I assume that inequality in local settings necessarily involves rich and poor living side by side, i.e. they are forced in local contexts to be in contact with one another, whereas inequality in more aggregated settings may involve economic segregation to a degree that means rich and poor do not typically come in contact with or observe one another. But of course there may be local areas that are completely economically segregated, and therefore local segregation is likely to be an important factor. I partially address this in the empirical analysis below by including a control for local economic segregation (available for the UK sample).*”**

**The addition of the control variable for economic segregation (taken as the multigroup entropy index) does not affect the estimates but is generally an important factor affecting pro-social behaviour in the expected direction (i.e. negative relationship). See Table 2, p.16 and Tables A.5-A.9.**

I also wonder how different measures of inequality affect the result and how the author deals with the high level of correlation between inequality and other (control) variables in the analysis. Does this affect the results?

**Robustness section includes alternative measures of inequality – please see Tables A.3 and A.5 in the Annex.**

**Regarding the correlation between inequality and controls, I have now provided a column in each of the main results tables with only inequality, income and the interaction between them as right-hand side variables. Please see Tables 1 and 2. The coefficients on the interaction term remains similar as when the controls are introduced, albeit the absolute size is reduced somewhat.**

5) Data: The author uses aggregated macro data from the US as well as a combination of macro & micro data from the UK. I am not entirely sure how the results of the different studies build up on each other and can be compared. Therefore, I urge the author to carefully portray the value of each study for the study and the overall research question(s)/testing of hypotheses once they are clearly specified.

**Thank-you for the opportunity to clarify this point, which is important. The UK study builds on the US one (as you point out, the US is not ‘micro’ in the sense that the observations are income groups). Thus I argue that the UK sample provides value (beyond being a different context for which to examine the local inequality hypothesis) by providing individual-level observations, thereby overcoming concerns around the ecological fallacy. I note this (last paragraph p.8) before introducing the UK study as a means to alleviate these issues: “*The income groups provided by IRS data are top coded and coarse, with households subsumed into income groups. The findings here might therefore be prone to the ecological fallacy [@kramer1983; @openshaw1984], whereby associations observed at the group level are different from that of individuals*”.**

6) Methods: More information and justification is needed for the selection of variables, use of methods and the statistical analyses – this would certainly benefit the paper. For example, why is the logarithmic function of the DV preferred over a linear one (p.5). What is the number of observations for the US? Why is the mediation analysis/variable not described in section 2? Do results vary with fixed effects for region?

**I take the logarithm of the percent income donated outcome measure (US sample) because this is strictly positive and rightward skewed, so interpretation is facilitated over the linear model (and the linear model not the most appropriate given it would extrapolate beyond the range of the data). I also take the logistic functional for the percentage of returns donating since the range of data is strictly 0 to 1 and so the linear model is again not the most appropriate (extrapolates beyond the range of the data). I’ve included a sentence to this effect (second paragraph p.8): “*I take the logistic functional form for the percentage of returns donating per income group since it varies between 0 and 1 (with the number of returns per group as weights), and I logarithmise the percent of income donated conditional on at least some households in the income group donating since the values are strictly positive and the distribution is skewed rightward.*”.**

**In practice this doesn’t make a difference, and I’ve indicated in a footnote that the results are unaffected if taking alternative functional forms, i.e. linear model for both main DVs for US sample (footnote 6, p.8): “*Using simple linear models rather than taking the logistic functional form or logarithm of the dependent variable does not affect the results (unreported).*”**

**I have decided to remove the section and discussion on mediation, as I do not feel that the analysis provides enough of a contribution to the paper overall and, as pointed out by editor, the assumptions for statistical mediation are unlikely to be met given the nature of the data.**

7) Choice of figure and tables: I advise that figures and tables should be carefully chosen to illustrate the main results. Overall, I found the tables more enlightening than most of the figures included in the paper. For tables I recommend the following: clear labelling of variables, clear specification of reference groups (for categorical variables), and the display of the constant (which is of high value for the interpretation of interaction effects).

**Thank-you, this is a useful point and I have removed the coefficient plots in favour of regression tables. All tables now all include constants** **and key reference groups are indicated in the notes of the tables (for the income group in US sample) and in the text.**

8) Interpretation of research findings: to allow a general readership to understand the value of the results, the interpretation of interaction effects could be more carefully described. At the same time, more careful description is required with regard to the different outcome variables (US vs. UK; absolute vs. relative donation) and their comparability. I also recommend differentiating between direct and indirect effects in the mediation analysis.

**Thank-you, I have spelled out the outcome measures more clearly, e.g. first paragraph of the methods section (middle p.7): “*Across both these samples, I focus on two outcome measures: 1) the amount donated to charity conditional on having donated something (i.e. the intensive margin), and 2) the likelihood of donating at all (i.e. the extensive margin).*”**

**Moreover, I have provided more interpretation of the relevant coefficients. See for example the first two paragraphs of the results section (from last paragraph p.10), and also the introduction which gives a preview of the results and makes clear the focus and findings across both samples.**

9) Language: the author often uses a very technical language throughout the paper (e.g. ‘negative interaction’, ‘opposite signs’, see paragraph 1 of the introduction for illustration). This should be avoided so to make this study also attractive and understandable for a larger readership. At the same time, the paper would benefit from more precision and clarity in the use of language with regard to the conceptual linkages between inequality and prosocial behaviour and its variation with household and personal characteristics (such as income and friendships).

**Thank-you for these suggestions – I am very keen for this article to be accessible to a large readership. I have ameliorated the language, especially in the introductory and concluding sections. For example, in the second paragraph p.2, I replaced “*interacts negatively with income*” to “*reduces pro-social behaviour among the rich*”.**

**Reviewer #3:**

Reading the article causes some dissonance. On the one hand, sophisticated analyses have been conducted using multi-level modeling. But on the other hand, the description is very narrative, in the style of a sociological essay.

The generally accepted rules about the content of the various parts of the manuscript are broken. In particular, methods appear everywhere - in the introduction, in the methodological section (rightly so here) and in the presentation of results. A more formalized form of method description (data sources, samples, dependent and independent variables, statistical methods) would make it easier for a large part of the audience to understand the results.

**Thank-you for this comment, which is very important as the main objective doing this work is to have others understand the results and (hopefully) as a result find them useful and/or build upon them. I have clarified the structure, for example confining the detail of the methods and data to that section (thereby including less detail in the introductory section). I have also provided a short summary of results in the introduction as is conventional, and included a paragraph laying out the structure at the end of the introduction (last paragraph, p.4). Finally, I move much of the description of the robustness checks to the methods section rather than have it in the results section.**

It is also surprising that the results except for one table are included in the appendix. It is difficult for me to judge whether this material will be available only electronically. I suggest selecting key results from Study 1 and Study 2 and including at least one core table or graph in each subsection (UK and US data, respectively).

**Thank-you, I have now included the full regression tables for main analysis in the body of the paper instead of the coefficient plots. Only the tables for the robustness checks are included in the Annex.**

Other suggestions are as follows:
- making the aim of the study more explicit at the end of the introduction instead of a paragraph about the analyses performed

**The introduction has now been substantially revised, with a clearer focus of the aims of the paper (testing the moderating effect of local inequality on the relationship between income and pro-sociality) and a preview of the results.**

- clear distinction of the description of the sample, variables and methods of analysis in Chapter 2 (divided into Study 1 and Study2) , while limiting this type of information in the introduction and results as already highlighted above

**Thank-you, the methods section has now been divided into two (US and UK studies respectively, and there is only limited information in the introductory and results sections on the methods and data.**

- marking the sources of data under the tables and explaining the abbreviations

**Variable labels should now be clear, and the sources are included in the notes at the bottom.**

- checking the clarity of the tables (for example, what is the difference between models 1 and 2 and 3 and 4); perhaps explanatory information can be in the title

**Thank-you, the title for the main tables should now be self-explanatory, and all the differences between columns explained in text or clear from the column headers.**

- verify that all tables and figures are assigned to US or UK populations (fig.3?).

**This should now be clear (titles have been homogenised) for the figures (Figures 2 and 3).**

**Annex**

**Table 1: Additional robustness check – removing ZIP-area observations according to size of Gini estimate margin of error**

|  | *Dependent variable:* | | | |
| --- | --- | --- | --- | --- |
|  |  | | | |
|  | ME < 90th percentile | ME <= 0.05 | ME < 90th percentile | ME <= 0.05 |
|  | Proportion donated | | Donation amount | |
|  | (1) | (2) | (3) | (4) |
|  | | | | |
| Inequality | 0.201^***^ | 0.264^***^ | -0.060^***^ | -0.093^***^ |
|  | (0.009) | (0.009) | (0.006) | (0.007) |
| $37,500 | 1.367^***^ | 1.355^***^ | 0.464^***^ | 0.486^***^ |
|  | (0.002) | (0.002) | (0.006) | (0.006) |
| $62,500 | 2.331^***^ | 2.313^***^ | 0.868^***^ | 0.897^***^ |
|  | (0.002) | (0.002) | (0.005) | (0.005) |
| $87,500 | 2.759^***^ | 2.759^***^ | 1.035^***^ | 1.077^***^ |
|  | (0.002) | (0.002) | (0.006) | (0.006) |
| $150,000 | 3.336^***^ | 3.328^***^ | 1.357^***^ | 1.420^***^ |
|  | (0.002) | (0.002) | (0.005) | (0.005) |
| $200,000+ | 4.275^***^ | 4.272^***^ | 1.895^***^ | 1.963^***^ |
|  | (0.002) | (0.002) | (0.006) | (0.006) |
| Median income | 0.150^***^ | 0.191^***^ | 0.172^***^ | 0.155^***^ |
|  | (0.014) | (0.012) | (0.008) | (0.008) |
| Ln(Population) | -0.036^***^ | -0.034^***^ | -0.041^***^ | -0.035^***^ |
|  | (0.007) | (0.005) | (0.004) | (0.004) |
| White (%) | -0.358^***^ | -0.313^***^ | -0.139^***^ | -0.155^***^ |
|  | (0.008) | (0.007) | (0.005) | (0.004) |
| Poor (%) | -0.282^***^ | -0.282^***^ | 0.043^***^ | 0.042^***^ |
|  | (0.011) | (0.010) | (0.006) | (0.006) |
| 25+ with college degree (%) | 0.318^***^ | 0.165^***^ | -0.093^***^ | -0.037^***^ |
|  | (0.012) | (0.011) | (0.007) | (0.007) |
| Age 65+ (%) | -0.006 | -0.009 | 0.186^***^ | 0.186^***^ |
|  | (0.008) | (0.007) | (0.004) | (0.004) |
| Age less than 18 (%) | -0.004 | -0.020^**^ | 0.027^***^ | 0.045^***^ |
|  | (0.009) | (0.008) | (0.005) | (0.005) |
| Inequality:$37,500 | -0.034^***^ | -0.046^***^ | 0.018^***^ | 0.027^***^ |
|  | (0.002) | (0.002) | (0.006) | (0.006) |
| Inequality:$62,500 | -0.057^***^ | -0.069^***^ | 0.041^***^ | 0.051^***^ |
|  | (0.002) | (0.002) | (0.006) | (0.006) |
| Inequality:$87,500 | 0.004^**^ | 0.001 | 0.081^***^ | 0.097^***^ |
|  | (0.002) | (0.002) | (0.006) | (0.006) |
| Inequality:$150,000 | 0.035^***^ | 0.029^***^ | 0.081^***^ | 0.099^***^ |
|  | (0.002) | (0.002) | (0.006) | (0.006) |
| Inequality:$200,000+ | 0.144^***^ | 0.139^***^ | 0.146^***^ | 0.173^***^ |
|  | (0.002) | (0.002) | (0.006) | (0.006) |
| Constant | -5.036^***^ | -4.898^***^ | -5.233^***^ | -5.353^***^ |
|  | (0.041) | (0.036) | (0.023) | (0.023) |
|  | | | | |
| State fixed effect | Y | Y | Y | Y |
| Random effect level | ZIP-code | ZIP-code | ZIP-code | ZIP-code |
| Observations | 125,862 | 97,431 | 78,260 | 68,685 |
| Log Likelihood | -1,021,974.000 | -814,024.300 | -53,463.050 | -42,450.840 |
| Akaike Inf. Crit. | 2,044,087.000 | 1,628,189.000 | 107,068.100 | 85,043.680 |
|  | | | | |
| *Note:* | ^*^p<0.1; ^**^p<0.05; ^***^p<0.01 | | | |
